# Production of 3,4-dihydroxy L-phenylalanine by a newly isolated *Aspergillus niger *and parameter significance analysis by Plackett-Burman design

**DOI:** 10.1186/1472-6750-10-86

**Published:** 2010-12-10

**Authors:** S Ali, I Haq

**Affiliations:** 1 Institute of Industrial Biotechnology (IIB), GC University Lahore, Lahore-54000, Pakistan

## Abstract

**Background:**

The amino acid derivative 3,4-dihydroxy L-phenylalanine (L-dopa) is gaining interest as a drug of choice for Parkinson's disease. *Aspergillus oryzae *is commonly used for L-dopa production; however, a slower growth rate and relatively lower tyrosinase activity of mycelia have led to an increasing interest in exploiting alternative fungal cultures. In the present investigation, we report on the microbiological transformation of L-tyrosine to L-dopa accomplished by a newly isolated filamentous fungus *Aspergillus niger*.

**Results:**

The culture *A. niger *(isolate GCBT-8) was propagated in 500 ml Erlenmeyer flasks and the pre-grown mycelia (48 h old) were used in the reaction mixture as a source of enzyme tyrosinase. Grinded mycelia gave 1.26 fold higher L-dopa production compared to the intact at 6% glucose (pH 5.5). The rate of L-tyrosine consumption was improved from 0.198 to 0.281 mg/ml. Among the various nitrogen sources, 1.5% peptone, 1% yeast extract and 0.2% ammonium chloride were optimized. The maximal L-dopa was produced (0.365 mg/ml) at 0.3% potassium dihydrogen phosphate with L-tyrosine consumption of 0.403 mg/ml.

**Conclusion:**

Over ~73% yield was achieved (degree of freedom 3) when the process parameters were identified using 2k-Plackett-Burman experimental design. The results are highly significant (p ≤ 0.05) and mark the commercial utility (LSD 0.016) of the mould culture which is perhaps the first ever report on L-dopa production from *A. niger*.

## Background

The 3,4-dihydroxy L-phenylalanine (L-dopa) is known to be produced from L-tyrosine by a one-step oxidation reaction under submerged batch culture [1]. The optimisation of cultural conditions is necessary for a successful cultivation process. The key enzyme responsible for biosynthesis of L-dopa is tyrosinase [2]. Tyrosinases are widely distributed and highly purified enzymes, derived from microbial (*Aspergillus, Rhizopus *and *Neurospora *spp.) or plant sources (*Agaricus *and *Vicia *spp.). However in microorganisms, enzyme activity is generally very weak; L-tyrosine along with L-dopa is rapidly decomposed to other metabolites. Thus, stoichiometric formation of L-dopa is difficult to achieve [3,4]. In addition, L-dopa is an unstable product in the reaction mixture and is further converted into dopaquinone and the final product melanin [5]. Because of the higher production cost and its greater commercial value, many researchers have investigated on the alternative production of L-dopa [6,7]. Investigations have now centred upon microbiological L-dopa production from *Erwinia herbicola *and *Escherichia coli *[8,9]. However, the production could be expensive due to the removal of proteins and hormones which would also be produced by the microbial cells. Another alternative method is the L-dopa production from L-tyrosine with an immobilized tyrosinase. The enzyme inhibits below pH 3.5 and thus decreases L-dopa production [10]. The optimal production was obtained when glucose was used as a carbon source [11,12]. The nitrogen sources as well as the concentration of nitrogen containing salts, sucrose and phosphate in the culture medium were found to greatly affect the biosynthetic pathway of L-dopa [13].

*Aspergillus oryzae *has largely been exploited as an organism of choice for L-dopa production; however its use has been limited drastically due to the strong tyrosinase inhibitors produced by the pre-grown mycelia [14]. In addition, a much slower growth rate of this fungus has urged to find a better alternative microorganism [5,15]. Therefore, in the present study, different strains of *A. niger *were isolated from bread wastes. Among them isolate GCBT-8 was found to be a faster growing culture and gave the highest product yield. An increase in biomass of this mould culture was attempted to further enhance L-dopa production under submerged cultivation. As tyrosinase is an intracellular enzyme, so mould mycelia were used for the biochemical conversion of L-tyrosine to L-dopa. The 2-factorial Plackett-Burman experimental design was used to further identify the significant variables influencing L-dopa production.

## Methods

### Maintenance of *A. niger*

Various strains of *A. niger *were isolated from bread wastes by pour plate method [5]. The samples were collected in sterilized polythene bags from the local market of Lahore (Pakistan). The strains were maintained on potato dextrose agar (PDA) medium, pH 5.6 and incubated at 30°C for 4-6 days until maximal sporulation. Preliminary screening of fungal isolates was accomplished using PDA medium containing 0.1% L-tyrosine as an inducer and bromocresol green dye as an indicator [5].

### Inoculum Preparation

The spore suspension was prepared by adding 10 ml of sterilized distilled water aseptically to a 4-6 day old slant culture having profuse growth on its surface. An inoculum needle was used to disrupt the clumps of spores. The tube was shaken gently to form a homogeneous suspension. The spore count was made on a haemocytometer.

### Propagation, Harvesting and Ultrasonication of Fungal Mycelia

The propagation of mycelia was carried out by taking 100 ml of medium containing 4 g/l glucose, 2 g/l peptone, 0.6 g/l NH_4_Cl, 0.6 g/l KH_2_PO_4_, 0.04 g/l MgSO_4_.7H_2_O, 2 g/l yeast extract at pH 5 in 500 ml Erlenmeyer flasks. The flasks were cotton plugged and sterilized in an autoclave at 15 lbs/in^2 ^pressure (121°C) for 20 min. The medium was inoculated with 5 ml of spore suspension (1.45×10^6 ^CFU/ml) of *A. niger*. The flasks were incubated at 30°C for 48 h in a rotary shaker (350 rpm). The mycelia were harvested by filtering through a funnel and washed free of adhering medium with ice cold water (4°C). These intact mycelia were dried in filter paper folds. The grinded form of mycelia was obtained after disrupting them by an ultrasonicator for 5 min [12]. Both the intact and grinded mycelia were stored at 4°C in a cold cabinet. All the experiments were run parallel in triplicates.

### Reaction Procedure and Critical Phases

The reaction for L-dopa production from L-tyrosine was carried out in a suspension of intact fungal mycelia [1]. The reaction mixture was prepared by adding 0.0625 g/l L-tyrosine, 1.875 g/l mycelia (intact or grinded), 0.125 g/l L-ascorbic acid in 50 mM acetate buffer (pH 3.5). Twenty five millilitre of this reaction mixture was taken in 250 ml Erlenmeyer flasks. The reactions were performed aerobically at 50°C for 60 min in a shaking water bath (240 rpm).

### Analytical Techniques

The sample was withdrawn and centrifuged at 9000×*g *for 15 min. The supernatant was used for analysis of L-dopa produced and L-tyrosine consumed in the reaction mixture following the analytical methods of Arnow [16].

### Estimation of L-Tyrosine

One millilitre of supernatant from the reaction mixture along with 1 ml of mercuric sulphate reagent was taken in a test tube. The tube was placed in a boiling water bath for 10 min. The assay mixture was cooled and 1 ml of nitrite reagent was added. The volume was raised upto 16 ml with distilled water. The diluted mixture was analyzed (A_550 nm_) by a spectrophotometer and the amount of residual L-tyrosine was determined after comparing with the tyrosine-standard.

### Estimation of L-Dopa

**Figure 1:**
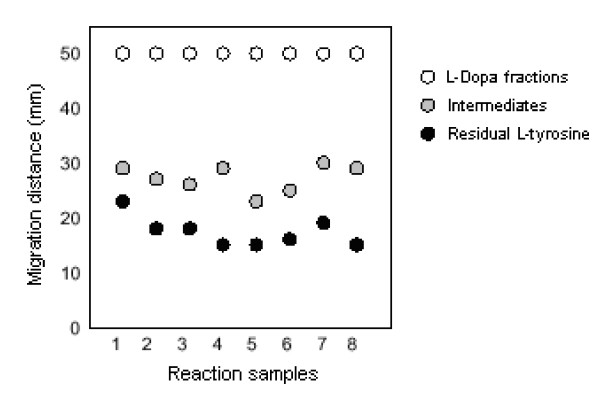
**Separation of L-dopa fractions from eight different samples of the reaction mixture run on paper chromatogram**.

One millilitre of supernatant from the reaction mixture was taken in a test tube. Then 1 ml of 0.5N HCl along with 1 ml of nitrite molybdate reagent was added to it. A yellow colour appeared. One millilitre of 1N NaOH was also added which gave a red coloration. The volume was raised upto 16 ml with distilled water. The diluted mixture was analyzed (A_550 nm_) by a spectrophotometer and the amount of produced L-dopa was determined after comparing with the dopa-standard. All preparations of L-dopa were also examined by infrared spectroscopy and further confirmed by paper chromatography [6]. The chromatography was performed on a Whatman filter paper (No. 1) using l-butanolacetic acid-distilled water (4:1, v/v) as a solvent. The chromatograms were developed with a equal volume mixture of 20 mM NaH_2_PO_4_, 50 mM acetonitrile and 35 mm L-catechol. Figure 1 shows a paper chromatogram, run for the separation of L-dopa fraction from residual L-tyrosine and other intermediates.

### Determination of Tyrosinase Activity

Tyrosinase activity was determined after Kandaswami and Vaidyanathan [17]. One unit of enzyme activity is equal to a ΔA_265 nm _of 0.001 per min at pH 6.5 (25°C) in a 3 ml reaction mixture containing L-catechol and L-ascorbic acid. For this, 2.6 ml of 50 mM potassium phosphate buffer along with 0.1 ml L-catechol, 0.1 ml L-ascorbic acid and 0.1 ml EDTA were mixed by inversion and equilibrated to 25°C. The ΔA_265 nm _was monitored until constant, followed by the addition of 100 μl of reaction broth. The decrease in ΔA_265 nm _was recorded for 5 min with an equal interval of 1 min. The ΔA_265 nm _was obtained using the maximal linear rate for both the test and control. Enzyme activity was determined using the following formula,

$$\text{Tyrosinase activity }(\text{U}/\text{mg})=\frac{\Delta {\text{A}}_{265\text{nm}}/\text{min test}-\Delta {\text{A}}_{265\text{nm}}/\text{min control}}{0.001\text{ mg enzyme}/\text{reaction mixture}}$$

### Kinetic Depiction

The kinetic parameters for L-dopa production and L-tyrosine consumption were studied according to the procedures of Pirt [18]. The volumetric rates for substrate utilization (Q_s _mg/ml/min) and product formation (Q_p _mg/ml/min) were determined from the maximum slopes in plots of substrate utilized and L-dopa produced each vs. the time of reaction. The product yield coefficient Y_p/s _was determined using the relationship i.e., Y_p/s _= dP/dS (mg/ml). The specific rate constants for product formation (q_p _mg/mg cells/min) and substrate utilization (q_s _mg/mg cells/min) were determined by the equations i.e., q_p _= μ×Y_p/x _and qs = μ×Y_s/x_, respectively.

### Statistical Analysis and Application of 2k-Factorial Design

Duncan's multiple range tests (Spss-16, version 11.5) were applied under one-way analysis of variance (I-ANOVA) and the treatment effects were compared according to Snedecor and Cochran [19]. Significance is presented in the form of probability (p ≤ 0.05) values. The significant variables were identified using a 2k-factorial system i.e., Plackett-Burman experimental design [20]. The variables were denoted at two widely spaced intervals and the effect of individual parameters on L-dopa production was calculated by the following equations,

(I) $${\text{E}}_{\text{o}}=(\Sigma {\text{M}}_{+}-\Sigma {\text{M}}_{–})/\text{N}$$

(II) $$\text{E}=\beta_{\text{1}}+\Sigma \beta_{\text{2}}+\Sigma \beta_{\text{3}}+\beta_{\text{123}}$$

In Eq. I, E_ο _is the effect of first parameter under study while M+ and M- are responses of L-dopa production by the filamentous fungus. N is the total number of optimizations. In Eq. II, E is the significant parameter, β_1 _is the linear coefficient, β_2 _the quadratic coefficient and β_3 _is the interaction coefficient for process parameters.

## Results and Discussion

### Screening of Fungal Cultures for L-Dopa Production

In the present study, several strains of *A. niger *were isolated from bread wastes by serial dilution method. Initially fungal isolates were screened on the basis of larger reddish zones of L-tyrosine hydrolysis in the growth medium, indicated by the presence of bromocresol green dye. Among them, isolate GCBT-8 was found to be a faster growing culture and gave the highest product yield. An increase in biomass of this mould culture was attempted to further enhance L-dopa production under submerged cultivation. Initially, the culture (strain GCBT-8) was used for the propagation of mycelia in 500 ml Erlenmeyer flasks for L-dopa production. The fungus *A. niger *GCBT-8 is capable of producing tyrosinase with activity 43.28 U/mg in an acidic reaction mixture which transformed tyrosine and its derivatives to L-dopa as reported by Krishnaveni et al. [21]. The fungal strain was grown in a medium containing carbon sources, nitrogen sources, minerals, and other essential nutrients. To obtain optimal yield of L-dopa, it was imperative to add L-ascorbic acid in the reaction broth to prevent melanin formation. Since tyrosinase appears to be an inducible enzyme, its activity should be optimally increased to convert more L-tyrosine into L-dopa [13,22].

### Propagation of *A. niger *Mycelia

**Figure 2:**
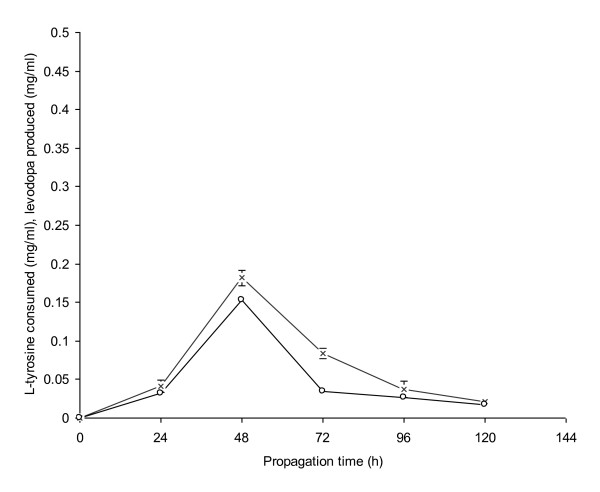
**Propagation of *A. niger *GCBT-8 for L-dopa production**. Glucose conc. 6% (w/v), initial pH 5. Standard bars indicate standard deviation (± sd) among the three parallel replicates (L-tyrosine consumed - × -, L-dopa produced - ○ -).

The maximum amount of L-dopa (0.1533 mg/ml) was produced when 48 h old mycelia were used in the reaction mixture as a source of enzyme tyrosinase (Figure 2). It was due to the increased tyrosinase activity of *A. niger *mycelia. A further increase in the time of mycelial propagation greatly reduced L-tyrosine consumption and subsequent L-dopa production possibly due to the conversion of L-dopa into other metabolites such as dopamine or dopachrome. The change in colour from red to black was due to the formation of melanin like substances in the reaction broth [23,24]. In the present study, at 96-120 h of propagation time, L-dopa production declined from 0.0263 to 0.173 mg/ml. Mason [25] reported that L-dopa production remained low in a short propagation period viz-a-viz a prolonged propagation time for mycelial cultivation greatly reduced L-dopa production. Melanosine and melanin were also produced in the reaction mixture but their highest volumetric productivities remained 0.015 and 0.0026 mg/ml, respectively.

### Effect of Initial pH

**Figure 3:**
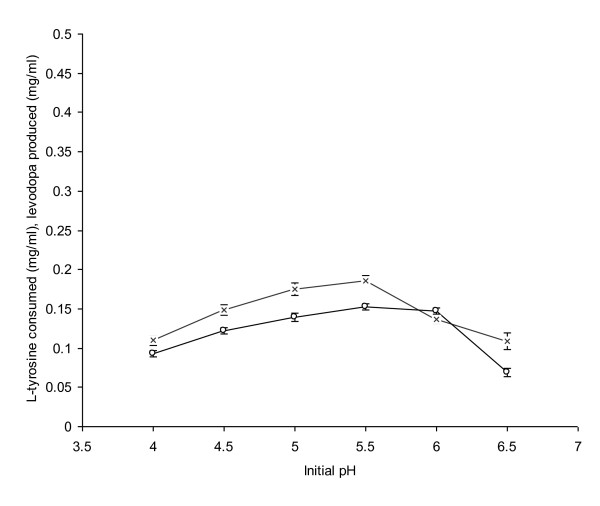
**Effect of different initial pH on the propagation of *A. niger *GCBT-8 for L-dopa production**. Glucose conc. 6% (w/v), propagation time 48 h. Standard bars indicate standard deviation (± sd) among the three parallel replicates (L-tyrosine consumed - × -, L-dopa produced - ○ -).

Initial pH has a profound effect on the bioconversion of L-tyrosine to L-dopa. The maximum L-dopa production (0.153 mg/ml) and consumption of L-tyrosine (0.186 mg/ml) was observed when pH of the cultivation medium was adjusted to 5.5 (Figure 3). It was due to the optimal growth of fungal mycelia because metabolic pathways were operating normally at this pH. The enzymes produced including tyrosinase, tyrosine hydroxylase and β-tyrosinase were also in their highest concentrations. These enzymes catalyzed the oxidation of tyrosine to a greater extent and a higher amount of L-dopa was thus produced in the reaction mixture. In a similar study, Haneda et al. [1] obtained maximum L-dopa production (0.084 mg/ml), when pH of the medium was adjusted to 5 rather at 5.5. A further increase in the pH of the medium other than the optimal greatly reduced both L-tyrosine consumption and L-dopa production. At pH ranging from 6-6.5 of the medium, L-dopa production declined from 0.147 to 0.0693 mg/ml due to the disturbed fungal physiology and tyrosinase depletion. However, Evan and Raper [26] reported the limits of tyrosinase activity at a broad pH range (5-10).

### Role of Intact and Grinded Mycelia Developed at Various Glucose Concentrations

**Figure 4:**
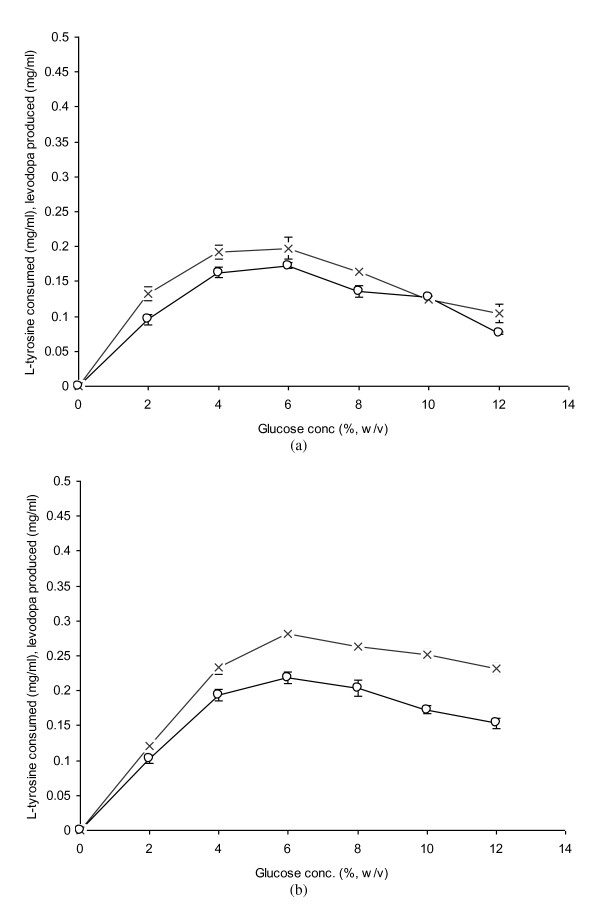
**Effect of glucose levels on the propagation of *A. niger *GCBT-8 for L-dopa production**. a) Intact mycelia, b) Grinded mycelia. Propagation time 48 h, initial pH 5.5. Standard bars indicate standard deviation (± sd) among the three parallel replicates (L-tyrosine consumed - × -, L-dopa produced - ○ -).

As tyrosinase is an intracellular enzyme, so mould mycelia were used for the biochemical conversion of L-tyrosine to L-dopa. A comparison for the effect of glucose concentration on the propagation of *A. niger *(intact and grinded mycelia) was made at glucose concentration ranging from 2-12% (w/v) for each set (Figure 4a &4b). L-Dopa production increased gradually from 2-6%. However, maximal L-dopa production (0.2183 mg/ml) was obtained with the grinded mycelia when 6% glucose as a basal carbon source was supplemented to the medium. The enzyme tyrosinase was restricted in the intact cells and didn't release into the reaction mixture. However in case of grinded mycelia, tyrosinase released into the reaction mixture catabolized the available L-tyrosine, hence resulted in the maximal L-dopa production. The consumption of L-tyrosine was recorded to be 0.281 mg/ml at the optimal glucose level. A gradual reduction in L-dopa production was observed with both the intact and grinded mycelia when glucose concentration was further increased other than the optimal. It was due to the accumulation of other derivatives like dopamine, dopaquinone or dopachrome. These results were substantiated with the findings reported by Sarin et al. [27] and Lee et al. [28]. An insignificant amount of L-dopa production (0.076 mg/ml) was however obtained with the intact mycelia at 12% glucose. L-tyrosine consumption was found to be 0.104 mg/ml. Therefore, grinded mycelia gave 1.26 fold higher L-dopa production compared to the intact at a glucose level of 6%.

### Evaluation of Nitrogen Sources

**Figure 5:**
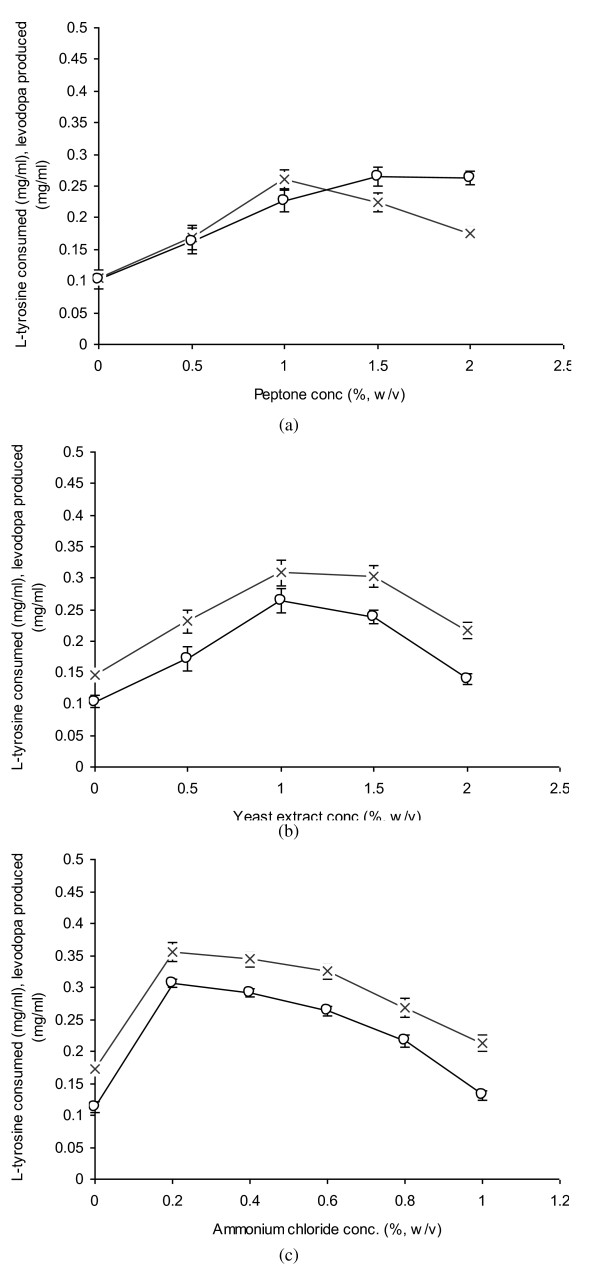
**Evaluation of nitrogen sources for the propagation of *A. niger *GCBT-8 for L-dopa production**. a) Peptone, b) Yeast extract, c) ammonium chloride. Glucose conc. 6% (w/v), propagation time 48 h, initial pH 5.5. Standard bars indicate standard deviation (± sd) among the three parallel replicates (L-tyrosine consumed - × -, L-dopa produced - ○ -).

The nitrogen source as well as its concentration in the cultivation medium greatly affects the biosynthesis of L-dopa from L-tyrosine. The optimal level of L-dopa production with 1.5% (w/v) peptone (0.265 mg/ml) and 1% (w/v) yeast extract (0.264 mg/ml) as organic nitrogen sources was recorded. The level of L-tyrosine consumption was noted to be 0.224 and 0.308 mg/ml, respectively (Figure 5a and 5b). L-Dopa production was however, declined when 0.5% (w/v) peptone (0.163 mg/ml) or 2% yeast extract (0.14 mg/ml) were added into the cultivation medium. It was due to the rapid biotransformation of tyrosine into L-dopa and then to dopachrome or indol-5,6-quinone. The final product was melanin with other pigmented intermediates as reported earlier [15,29,30]. Similar kinds of findings have also been reported by Huang et al. [31]. L-Dopa can be produced with higher efficiency using ammonium chloride as an inorganic nitrogen source. The maximum production (0.306 mg/ml) was observed at 0.2% ammonium chloride (Figure 5c). It was found optimal as tyrosinase activity was increased; hence a higher rate of L-dopa production was observed. A decreased level of L-dopa production (0.131 mg/ml) was possibly due to the lower mycelial growth which was not enough to oxidize L-tyrosine into L-dopa completely. L-Dopa production was almost doubled compared to other ammonium salts used solely in the medium. When ammonium chloride was used as source of ammonium ion, β-tyrosinase activity was more than 1.62 fold higher than that obtained with other sources of ammonium. The maximum L-dopa (0.134 mg/ml with 0.428 mg/ml L-tyrosine consumption) was achieved at 4% level of the optimal nitrogen source used [13].

### Evaluation of Potassium Sources

**Figure 6:**
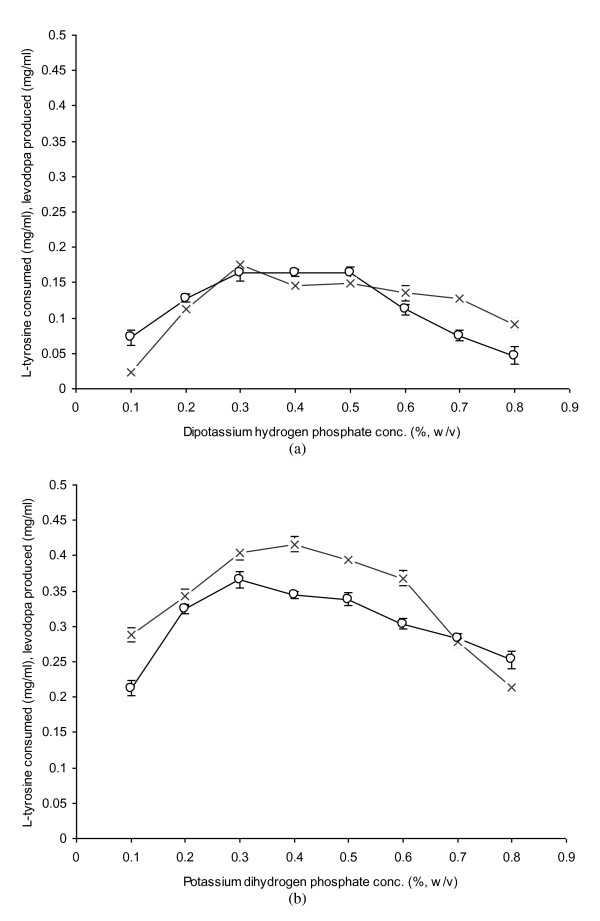
**Evaluation of potassium sources for the propagation of *A. niger *GCBT-8 for L-dopa production**. a) Dipotassium hydrogen phosphate conc., b) Potassium dihydrogen phosphate conc. Glucose conc. 6% (w/v), propagation time 48 h, initial pH 5.5. Standard bars indicate standard deviation (± sd) among the three parallel replicates which differ significantly at p ≤ 0.05 (L-tyrosine consumed - × -, L-dopa produced - ○ -).

The effect of potassium sources viz. potassium dihydrogen phosphate and dipotassium hydrogen phosphate for L-dopa production from L-tyrosine in the reaction mixture by *A. niger *was also investigated. The concentration was ranged from 0.1 to 0.8 mg/ml for each trial (Figure 6a &6b). The maximum L-dopa production (0.365 mg/ml) was obtained at 0.3% (w/v) potassium dihydrogen phosphate with L-tyrosine consumption of 0.403 mg/ml. L-Dopa production was gradually decreased at higher concentrations of potassium dihydrogen phosphate other than 0.3%. The production declined to 0.253 mg/ml when 0.8% potassium dihydrogen phosphate was added into the cultivation medium. Potassium helped the mycelial cells to grow faster with a much better vigour. This element also produced a fluffy mass of mycelia which liberated the enzyme tyosinase in larger quantities when treated in L-ascorbate acidulated reaction broth as reported by Kuo et al. [32]. The addition of dipotassium hydrogen phosphate was, however not found significant for both L-tyrosine consumption and L-dopa production at all the rates examined. The level of product formation (0.047-0.164 mg/ml with a decreased rate of substrate consumption from 0.023 to 0.176 mg/ml) remained all the way less compared to the lowest concentration of potassium dihydrogen phosphate (0.212 mg/ml L-dopa at 0.1%).

### Kinetic Study

The kinetic variables notably product formation parameters (Q_p_, Y_p/s_, q_p_) and substrate consumption parameters (Q_s_, q_s_) by *A. niger *GCBT-8 were compared for intact and grinded mycelia as sources of enzyme tyrosinase, β-carboxylase or tyrosine hydroxylase (Table 1). The comparison of Q_p _(mg/ml/min) for L-dopa productivity demonstrated that the grinded mycelia have a higher value for volumetric rate of product formation (0.0061 mg/ml/min) than the intact mycelia. Several fold improvement in terms of volumetric productivity was noted with the grinded mycelia at all the rates examined. In addition, when both of the mycelia were monitored for specific rate constant, the former gave relatively higher values for q_p _than the use of intact mycelia in the reaction mixture. The grinded mycelia exhibited an overall 4-6 fold improvement in the values for Q_p_, Y_p/s _and q_p _compared to the intact (LSD 0.016) which is highly significant (*HS*, *p *≤ 0.05) and it was further supported by the findings reported by Pirt [18]. Haq et al. [15] found that nutritional parameters influence the substrate consumption rate, specific growth and subsequent productivity of tyrosinases. It is evident from the kinetic values that the enzymes are intracellular rather than extracellular and these have higher affinity for tyrosine as a substrate compared to other amino acids or derivatives of L-tyrosine. Haq and Ali [7] attempted to hyper produce L-dopa by 0.16 μg vermiculite addition during reaction and achieved 0.055 mg/ml of the actual product required. In the present study, maximum Y_p/s_, Q_p _and q_p _were several-fold improved over those from some other *Aspergillus *or *Cellulomonas *spp. [12]. As L-dopa is a high cost but low yield product; the maximal L-dopa production from the amino acid i.e., L-tyrosine was attributed to the medium composition, cultivation design, tonicity of the reaction broth and mycelial morphology of the culture used [4,18].

**Table 1:** Comparison of kinetic variables for propagation of A. niger GCBT-8 for L-dopa production

Kinetic variables	L-dopa production (mg/ml)	
	**Intact mycelia**	**Grinded mycelia**

Product formation parameters		
Q_p _(mg/ml/min)	0.0029 ± 0.001^bc^	0.0061 ± 0.003^a^
Y_p/s _(mg/mg)	0.875 ± 0.03^abc^	0.905 ± 0.05^ab^
q_p _(mg/mg cells/min)	0.0012 ± 0.001^b^	0.0024 ± 0.0003^a^

Substrate consumption parameters		
Q_s _(mg/ml/min)	0.0033 ± 0.001^bcd^	0.0067 ± 0.003^ab^
q_s _(mg/mg cells/min)	0.0002 ± 0.0001^bc^	0.0003 ± 0.0001^ab^

LSD	0.059	0.016

<p>	HS	S

Kinetic variables: Q_p _(mg L-dopa produced/ml/min), Y_p/s _(mg L-dopa produced/mg L-tyrosine consumed), q_p _(mg L-dopa produced/mg cells used/min), Q_s _(mg L-tyrosine consumed/ml/min), q_s _(mg L-tyrosine consumed/mg cells used/min).

HS is for the 'highly significant' while S for 'significant' values. LSD is for least significant difference and <p> is for significance level in terms of probability. ± Indicates standard deviation among three parallel replicates. The values designated by different letters in each row differ significantly at p ≤ 0.05.

### Application of 2k-Plackett-Burman Design

A two way factorial experimental method i.e., 2k-Plackett-Burman design was applied to determine the significant process parameters for L-dopa production by *A. niger *GCBT-8. The data are given in Table 2. The validation of the model was investigated under the conditions predicted against the responses obtained for enhanced L-dopa production. A slightly differential correlation was observed between the observed and predicted values. The optimal levels of the significant process parameters for improved L-dopa production in shaking culture were time course (48 h), initial pH (5.5), use of grinded mycelia, optimal glucose level (6%, w/v), peptone (1.5%, w/v), yeast extract (1%, w/v), ammonium chloride (0.2%, w/v) and potassium dihydrogen phosphate (0.3%, w/v). The optimal tyrosinase activity was recorded to be 64.55 U/mg. The statistical analyses of the responses for L-dopa production were also performed and are represented in Table 3. The correlation (0.012*E*+0025), A, B, C_2 _and D for E values depicts that the model was highly significant (p ≤ 0.05). Correspondingly, the lower probability values also indicated that the model terms were significant. The analysis of linear, quadratic and interaction coefficients were performed on the fermentative results which highlighted that L-dopa production was a function of the independent parameters [20,33]. The addition of organic and inorganic nitrogen sources along with potassium source (degree of freedom 3) was found necessary for maintaining the spatial conformation of the enzyme tyrosinase, and thus has an important physiological role in the L-dopa activity and stability.

**Table 2:** Application of Plackett-Burman design at various process parameters (designated by different captions) for L-dopa production by A. niger GCBT-8

Process parameters at 2-factorial design						Tyrosinase activity (U/mg)	L-dopa production (mg/ml)	
		
Time course (h)^A^	Initial pH^B^	Glucose conc. (%, w/v)^C^_1_^+C^_2_		Ammonium chloride conc. (%, w/v)^D^	Potassium dihydrogen phosphate conc. (%, w/v)^E^		Observed	Predicted
							
		Intact	Grinded					
24	4.5	2	4	-	0.1	16.82	0.124	0.196
24	5	4	4	-	0.2	28.06	0.171	0.215
48	5	4	6	0.2	0.3	43.28	0.228	0.266
48	5.5	6	6	0.2	0.3	64.55	0.365	0.388
72	6	6	8	0.4	0.4	49.34	0.302	0.356

The different letters represent significant process parameters for L-dopa production. Statistical analysis of the model was based on 2-factorial experimental design. The dashes indicate the control experiments with no nutritional supplementation.

**Table 3:** Statistical analysis of 2-factorial experimental design at various significant process parameters for L-dopa production by A. niger GCBT-8

Significant process parameters	Sum mean values	F-value	Degree of freedom	Probability <p>
A	1.364	0.242	1	0.087
B	1.925	0.565	1	0.076
C_1_	1.981	0.804	1	0.069
C_2_	2.186	1.082	2	0.062
D	2.862	1.243	2	0.048
E	3.695	1.426	3	0.026
Correlation	0.012*E*+0025			

CM - 19.24; *R^2 ^*- 0.238. The letters represent significant process parameters (time course, initial pH, glucose levels by intact and grinded mycelia, inorganic and organic nitrogen sources, potassium sources) for L-dopa production.

## Conclusion

Tyrosinase is an intracellular enzyme so mould mycelia of a locally isolated *A. niger *GCBT-8 were used for the biochemical conversion of L-tyrosine to L-dopa in an ascorbate-acidulated reaction mixture. The maximal L-dopa (0.365 mg/ml) was found at L-tyrosine consumption of 0.403 mg/ml. The process parameters particularly nature of the carbon-limiting substrate, medium composition and nutritional supplements for L-dopa production were determined using Plackett-Burman design. The correlation (0.012*E*+0025), A, B, C_2 _and D for E values depicts that the model terms were highly significant (p ≤ 0.05). However, further work on the optimization of mycelial and L-tyrosine concentration is in progress prior to scale up studies.

## Abbreviations

L-dopa: 3,4-dihydroxy L-phenylalanine; rpm: revolutions per minute; Q_p_: mg L-dopa produced/ml/min; Y_p/s_: mg L-dopa produced/mg L-tyrosine consumed); q_p_: mg L-dopa produced/mg cells used/min; Q_s_: mg L-tyrosine consumed/ml/min); q_s_: mg L-tyrosine consumed/mg cells used/min; HS: highly significant; S: significant; LSD: significant difference; <p> significance level in terms of probability.

## Competing interests

The authors declare that they have no competing interests.

## Authors' contributions

SA conceived of the study and performed the experimental work. HI helped in the critical review and provided necessary facilities. The authors read and agreed to the final manuscript.
